# Calculated parenteral initial treatment of bacterial infections: Respiratory infections

**DOI:** 10.3205/id000059

**Published:** 2020-03-26

**Authors:** Sebastian R. Ott, Klaus-Friedrich Bodmann, Béatrice Grabein, Gert Höffken, Martin Kolditz, Hartmut Lode, Mathias W. Pletz, Florian Thalhammer

**Affiliations:** 1 Pneumologie/Thoraxchirurgie, Claraspital Basel, Switzerland; 2 Klinik für Internistische Intensiv- und Notfallmedizin und Klinische Infektiologie, Klinikum Barnim GmbH, Werner Forßmann Krankenhaus, Eberswalde, Germany; 3 Stabsstelle Klinische Mikrobiologie und Krankenhaushygiene, Klinikum der Universität München, Munich, Germany; 4 Dresden, Germany; 5 Pneumologie, Universitätsklinikum Carl Gustav Carus, Dresden, Germany; 6 Research Center Charlottenburg, Berlin, Germany; 7 Institut für Infektionsmedizin und Krankenhaushygiene, Universitätsklinikum Jena, Germany; 8 Klinische Abteilung für Infektiologie und Tropenmedizin, Medizinische Universität Wien, Vienna, Austria

## Abstract

This is the fifth chapter of the guideline “Calculated initial parenteral treatment of bacterial infections in adults – update 2018” in the 2^nd^ updated version. The German guideline by the Paul-Ehrlich-Gesellschaft für Chemotherapie e.V. (PEG) has been translated to address an international audience.

It provides recommendations for the empirical and targeted antimicrobial treatment of lower respiratory tract infections, with a special emphasis on the treatment of acute exacerbation of COPD, community-acquired pneumonia and hospital-acquired pneumonia.

## Zusammenfassung

Dies ist das fünfte Kapitel der von der Paul-Ehrlich-Gesellschaft für Chemotherapie e.V. (PEG) herausgegebenen S2k Leitlinie „Kalkulierte parenterale Initialtherapie bakterieller Erkrankungen bei Erwachsenen – Update 2018“ in der 2. aktualisierten Fassung.

Es enthält Empfehlungen für die empirische und gezielte antimikrobielle Behandlung von Infektionen der unteren Atemwege mit besonderem Schwerpunkt auf der Behandlung bei akuter Exazerbation der COPD sowie ambulant und nosokomial erworbenen Pneumonien.

## Introduction

Respiratory infections are the most common infectious diseases in adults, both amongst in-patients and out-patients. Successful treatment of bacterial diseases is ensured by a rapid initiation of an appropriate antimicrobial treatment. In general, initial treatment is started empirically, since the causative pathogen is usually not yet known.

Viruses are the dominating pathogens of upper respiratory tract infections, whereas bacteria are predominant in lower respiratory tract infection. The most common bacterial pathogens in community-acquired respiratory infections are pneumococci. In addition, *Haemophilus* *influenzae*, *Moraxella* *catarrhalis*, *Mycoplasma* *pneumoniae*, Legionella and Enterobacteriaceae play a role. *Staphylococcus* *aureus* and *Chlamydophila* *pneumoniae* also occur rarely. For Germany, the CAPNETZ study has provided epidemiological data. Difficult to treat multidrug-resistant pathogens, especially methicillin-resistant *Staphylococcus aureus* (MRSA) and *Pseudomonas* spp. only play a minor role in community-acquired lower respiratory tract infections in Germany. An exception here are acute exacerbations in patients with severe COPD, with bronchiectasis or with known chronic infection with *Pseudomonas*.

The pathogen spectrum of nosocomial pneumonias is much broader and, in addition to the pathogens that also occur in community-acquired infections, more frequently includes multidrug-resistant pathogens such as MRSA, extended spectrum beta-lactamase (ESBL)-producing Enterobacteriaceae, *Pseudomonas aeruginosa*, *Acinetobacter* spp. and *Stenotrophomonas* *maltophilia*.

The resistance of pneumococci to penicillin remains favorable in Germany with a maximum of 2% resistant strains. The macrolides show a declining trend (see chapter 2 [1]). An up to date overview of the resistance status of the most common bacterial pathogens of lower respiratory tract infections can be found in the Resistance Study of the Paul-Ehrlich Society (https://www.p-e-g.org/resistenzdaten.html) and the Antibiotic Resistance Surveillance system (ARS) of the Robert Koch Institute (https://ars.rki.de).

The specific resistance situation of nosocomial pneumonia pathogens in Germany has not been investigated in any large-scaled epidemiological study. It is subject to variations between individual clinics and even between individual wards, so that knowledge of local epidemiology and susceptibility is of particular importance for the local implementation of treatment recommendations or guidelines.

The PEG resistance study indicates that the proportion of ESBL-producing Enterobacteriaceae has increased over the last 15–20 years. In the 2013 study, which included a total of 22.5% of respiratory isolates, the proportion of *Escherichia* *coli* was 14.9% and *Klebsiella* *pneumoniae* 17.4%.

Antibiotic treatment within 3 months prior to resumed treatment predisposes to infections by resistant pathogens, especially against the previously used drug group. This correlation is well documented for beta-lactams, macrolides and fluoroquinolones.

The present guideline complements the S3 guidelines on “Community-acquired Pneumonia” from 2016 and on “Nosocomial Pneumonia” from 2017. Both guidelines were developed in collaboration with the Paul-Ehrlich Society for Chemotherapy (PEG) e.V. In the following, therefore, references are deliberately made to these two S3 guidelines [2], [3]. However, the present guideline is not applicable to mucoviscidosis (cystic fibrosis, CF) patients with chronic *Pseudomonas aeruginosa* infection and only of limited use in bronchiectasis patients with chronic *Pseudomonas aeruginosa* infection. In these cases, we refer to the corresponding AWMF S3 guideline “Pulmonary Disease in Mucoviscidosis, Module 2: Diagnosis and treatment in chronic infections with Pseudomonas aeruginosa” [4] and the “European Respiratory Society (ERS) Guidelines for the Management of Adult Bronchiectasis” [5].

## Acute exacerbation of COPD (AECOPD)

### Definition of AECOPD

There are different definitions of AECOPD in the literature; a universal definition is missing. This makes it difficult to compare the available studies. For this guideline, the following definition is used for acute exacerbation of COPD (AECOPD): Acute worsening of respiratory symptoms in known chronic obstructive pulmonary disease (COPD), which requires intensive treatment beyond basic daily treatment.

### Etiology of AECOPD

Nearly half of all AECOPD episodes are triggered by infectious pathogens. These are predominantly respiratory viruses, such as Respiratory Syncytial Virus (RSV), rhino, corona- and adenoviruses, human metapneumovirus (hMPV) and influenza viruses.

The most common bacterial agents are *Haemophilus influenzae*, *Streptococcus pneumoniae*, *Moraxella catarrhalis* and Enterobacteriaceae. In patients with severe COPD, *Pseudomonas* *aeruginosa* is also found.

### Clinical symptoms of AECOPD

Cardinal symptoms of AECOPD are

- increasing shortness of breath,
- increased cough,
- increase of sputum volume and/or viscosity,
- chest tightness.

Unspecific symptoms are

- fatigue,
- sleep disorders,
- depression or
- impaired consciousness, coma (CO_2_ narcosis).

### Indications for antimicrobial treatment in moderate and severe AECOPD

Since most of the infection-induced AECOPD are caused by viruses, there is no indication or option for initiating antimicrobial treatment in these cases, except for influenza. In clinical practice, it is often difficult to distinguish between viral and bacterial exacerbations; mixed infections may also occur. Even if the clinical manifestations of AECOPD are non-specific, in such cases the presence of increased sputum purulence may be used in addition to the severity of AECOPD when deciding to initiate antimicrobial treatment. Furthermore, measurement of procalcitonin (PCT) in the serum may also be used in decisions about initiating antibiotic therapy, if available. If the PCT level in the serum is <0.1 ng/ml, antimicrobial therapy can usually be refrained from.

For out-patients, antibiotic treatment is rarely indicated and should be administered orally. In hospitalized patients, antimicrobial treatment is generally indicated only if purulent sputum is present (Stockley type II exacerbation) or if the severity of the exacerbation requires intensive care treatment, particularly if invasive or non-invasive mechanical ventilation is needed. 

Recommendations for calculated parenteral antibiotic treatment in AECOPD are given in Table 1 (Tab. 1).

Antibiotic therapy is generally recommended (recommendation grade B) in:

- Moderate AECOPD (with indication for hospitalization): antimicrobial therapy only in patients with Stockley type II exacerbation (purulent sputum). PCT measurement may be considered, if available. At serum levels of ≤0.1 ng/ml, antibiotics can be dispensed with, regardless of the presence of sputum purulence.
- Severe AECOPD (with indication for ICU treatment): antimicrobial therapy is always indicated.

## Pneumonia

Pneumonia is defined as a microbial infection of the lung parenchyma. Clinically, pneumonia is present when new or increasing infiltrates are detected on chest radiography and some of the following clinical signs are present:

- body temperature >38°C (or rarely <36°C),
- Leukocytosis (> 10 G/L)
- left shift (>5%)
- productive cough,
- purulent expectoration,
- dyspnea, tachypnea,
- chills,
- rales
- chest pain,
- new onset or progressive confusion.

Pneumonias are classified according to the place of acquisition and the immune status of the patient according to the so-called “pneumonia triad” (see Table 2 (Tab. 2)) [6].

Additionally, in 2005, the American Thoracic Society (ATS) and the Infectious Diseases Society of America (IDSA) introduced the concept of “healthcare associated pneumonia” (HCAP), which includes nursing home acquired pneumonia (NHAP). This concept was primarily developed to define a group of patients at high-risk for infections caused by multi-drug resistant organisms (MDROs) and treat them accordingly [7]. However, this concept has shown to be non-predictive of MDROs and implies a significant over-treatment [8], [9]. Thus, in clinical routine, it is appropriate to define individual risk factors for the presence of MDROs infections that may require an empirical CAP treatment, which is different from the recommended standard treatment regiments (see below).

In addition to the rapid initiation of an adequate calculated antimicrobial treatment, adjuvant measures are important in the management of CAP, as they may help to reduce lethality. Beside sufficient fluid resuscitation and treatment with vasopressors, particularly in patients with hypotension and/or septic shock, these adjuvant therapies include measures to reduce fever, supplementary oxygen in hypoxia, mechanical ventilation in respiratory failure and treatment of bronchial obstruction. Administration of systemic glucocorticosteroids is currently only recommended in patients with concomitant bronchial obstruction or septic shock. All CAP patients should receive thrombosis prophylaxis.

Furthermore, the recognition of potentially unstable comorbidities, in particular cardiovascular diseases, is of great importance, because in the context of acute systemic inflammation in pneumonia, a worsening of the underlying disease may occur with a significant increase in mortality [10].

## Community-acquired pneumonia (CAP)

The choice of empiric antibacterial treatment in patients with CAP depends primarily on the expected spectrum of pathogens, the severity of the disease and individual risk factors for the presence of multidrug-resistant pathogens (MDROs). 

In addition to the clinical assessment of severity (clinical judgement), appropriate scores can be used to assess objectively the individual mortality risk. In clinical routine, the CRB-65 score has proven to be a reliable tool. Additionally, the functional status should be assessed to identify patients at increased risk of an unfavorable course and a clinical evaluation of potentially unstable co-morbidities and oxygenation should be performed. In addition, the modified minor criteria (of the IDSA/ATS guidelines) should be used for risk stratification (see Table 3 (Tab. 3)), especially in the elderly where the predictive value of the CRB-65 score is limited through age.

### CRB-65 score

Check for the following criteria:

- impaired consciousness,
- respiratory rate ≥30/min,
- diastolic blood pressure ≤60 mm Hg/systolic blood pressure <90 mm Hg,
- age ≥65 years.

The score is calculated by adding one point for each of the listed criteria, if present. It must be noted that individual patients, despite their initial assignment to a low-lethality group, may in some cases in the short-term deteriorate dramatically and require intensive medical care, which is associated with a significantly increased mortality [11]. The modified minor criteria can help to detect these patients early.

The present guideline addresses medium and severe CAP, as mild CAP can usually be treated orally.

- Moderate community-acquired pneumonia: in-patient management, usually on a normal ward (hospitalized CAP)
- Severe community-acquired pneumonia: in-patient management, usually on a monitoring ward (intensive care unit, intermediate care, etc.) (severe CAP).

### Pathogen spectrum in hospitalized CAP patients

By far the most common pathogen is *Streptococcus* *pneumoniae*; less frequently *Mycoplasma pneumoniae*, *Haemophilus influenzae*, respiratory viruses and *Staphylococcus aure*us and even less frequently Enterobacteriaceae, *Chlamydophila* spp. and *Coxiella burnetii* [2], [12]. The frequency of *Legionella pneumophila* varies from region to region and reaches up to 6%. Enterobacteriaceae are detected slightly more frequently than in CAP patients who can be treated in an ambulatory care setting. In a recent publication, by using modern pathogen detection methods such as nucleic acid amplification tests (NAT) an increase has been described in the detection rate of respiratory viruses in patients with CAP, both as single pathogens or together with various other microorganisms. In this study, rhino and influenza viruses were the most commonly detected pathogens in hospitalized CAP patients, even ahead of pneumococci [13]. It should be noted, however, that despite extensive diagnostics efforts (in this study), the detection of pathogens was successful in only 38% of all patients and the majority of the patients were classified as having a low-lethality risk (“mild CAP”; 70% of all patients had a CURB-65 score of ≤1 and 65% of all patients were in PSI grades 1–3, respectively). In addition, the question of the clinical significance of these virus detections has not yet been conclusively proven, as even in asymptomatic patients pathogens, especially rhinoviruses can be detected by NAT in respiratory material [14], [15].

According to current data from CAPNETZ, *Pseudomonas aeruginosa* and other MDROs such as MRSA play only a minor role as pathogens of CAP in Germany. As a result, only in selected patients with individual risk factors for MDROs (see below) a respective initial treatment is required. 

In each case, the individual risk of an infection caused by MDROs should be evaluated regardless of (CAP) severity. Major risk factors for the presence of resistant or multidrug-resistant pathogens include previous acquisition, usually in the context of a previous hospital stay or previous selection, for example by antibiotic pre-treatment. In addition to the presence of a respective risk factor, the duration of the exposure is of importance (for instance length of hospitalization, duration of antibiotic treatment). 

### Risk factors for multidrug-resistant pathogens

- transmission of resistant pathogens (high: previous hospitalization, possible: dialysis, nursing home)
- previous antimicrobial treatment (depending on spectrum, duration and dosage)
- severe structural chronic lung diseases such as severe COPD, bronchiectasis or mucoviscidosis with prior antibiotic treatment or previous hospitalization within the past three months
- known colonization by MDROs (such as MRSA or MRGN)
- admission from long-term care facilities, chronic dialysis, tracheostoma, open skin wounds.

### Treatment in hospitalized CAP patients

Antimicrobial treatment should be initiated as soon as possible, especially in severe CAP. In patients with severe sepsis or shock, delayed treatment initiation is associated with increased lethality after only 1–2 hours. Diagnostic procedures should therefore never delay the initiation of treatment. Recommendations for the calculated initial therapy for moderate and severe CAP without risk factors for MDROs are given in Table 4 (Tab. 4). In general, parenteral administration should be preferred in hospitalized patients. The administration of macrolides as part of combination treatment can be carried out orally; however, modern macrolides (clarithromycin, azithromycin) should be preferred. If prior antibiotic treatment has been received within the past 3 months, a change of the drug class is recommended.

If possible, initial parenteral treatment should always be switched early to oral treatment (sequential therapy). This is usually possible if the following conditions (clinical stability) are present (recommendation grade A):

- heart rate ≤100/min,
- respiratory rate ≤24/min,
- systolic blood pressure ≥90 mm Hg,
- body temperature ≤37.8°C,
- maintain oral intake,
- normal mental state,
- no hypoxemia (PO_2_≥60 mmHg or SaO_2_≥90%) and
- safe oral medication.

In general, drugs with high to very high oral bioavailability and proven efficacy in pneumonia are suitable for oral sequential therapy. If possible, it should remain within the same drug classes. However, it is also possible to change the drug class if no oral formulation of the corresponding antibiotic is available. If the pathogen has not been identified at this point in time, oral sequential therapy should be carried out following Table 5 (Tab. 5). In the case of sequential therapy, the possibility of a de-escalation of the antibiotic treatment should always be checked once the pathogen has been identified. 

### Adjustment of initial therapy – targeted treatment and de-escalation

After receiving the results of the microbiological tests, the calculated initial treatment in patients with community-acquired pneumonia should be adjusted according to the results of identified pathogens and in vitro resistance testing. In general, de-escalation using antibiotics with a targeted and thus narrower spectrum of activity is possible. This can reduce selection pressure and potentially reduce microbiological collateral damage. For example, a change to penicillin G in case of proven pneumococcal CAP and corresponding susceptibility. In the case of clinical stabilization and lack of evidence of an atypical bacterial pathogen, additional macrolide treatment should be stopped after 3 days. Further options for targeted antimicrobial treatment of common CAP pathogens can be found in Table 6 (Tab. 6).

### Duration of treatment

Antibiotic treatment can be stopped 48–72 hours after clinical improvement with subsidence of fever but usually at the earliest after 5 days. Treatment for more than 7 days is usually not required. If infection with *Pseudomonas aeruginosa* is proven, a longer treatment period of 8–15 days is recommended.

For dosage recommendations, see Table 7 (Tab. 7).

## Management of severe community-acquired pneumonia (sCAP)

The indication for intensified monitoring (depending on the care facility, intensive or intermediate ward or intensified monitoring on a normal ward) applies in patients with

- need for mechanical ventilation or septic shock
- presence of at least 1 minor criterion (see Table 3 (Tab. 3))
- insufficient oxygenation at room air (SaO_2_<90%)
- unstable or decompensated comorbidities.

In any case, thorough clinical assessment of the CAP severity is necessary to decide on intensified care, including evaluation of potentially decompensated comorbidities and oxygenation.

An individualized rapid fluid therapy with crystalloid solutions as well as immediate initiation of antimicrobial treatment (if possible within one hour) are essential for these patients. Further treatment of sepsis should be based on the guidelines for sepsis. 

### Pathogen spectrum of sCAP

The etiology of sCAP differs from the less severe form; the spectrum of pathogens is wider. Around 10% of infections are polymicrobial.

In a differential treatment approach in sCAP, the question of a possibility of multidrug-resistant pathogens (MDROs), including a risk of *Pseudomonas aeruginosa*, is of greater importance.

### Treatment of severe community-acquired pneumonia (sCAP)

The risk of an unfavorable outcome resulting from inadequate treatment due to resistance is higher for sCAP. Considering the current resistance data is therefore of particular importance. In patients with septic shock and/or invasive mechanical ventilation, initial combination therapy including a beta-lactam is indicated.

For sCAP without risk of infection caused by MDROs, including *Pseudomonas aeruginosa*, the S3 guideline on community-acquired pneumonia recommends (recommendation grade B) combination therapy as the treatment of choice, consisting of an adequate beta-lactam antibiotic (cefotaxime, ceftriaxone, piperacillin/tazobactam, ertapenem) and a macrolide (Table 4 (Tab. 4)) [2]. Monotherapy with a pneumococcal fluoroquinolone (levofloxacin or moxifloxacin) is a possible alternative. However, this recommendation only applies to patients without septic shock or invasive mechanical ventilation. 

In patients with an indication for empiric treatment against MDROs, including *Pseudomonas aeruginosa*, combination therapy is usually recommended consisting of piperacillin/tazobactam, cefepime, imipenem or meropenem and a *Pseudomonas*-active fluoroquinolone (levofloxacin or ciprofloxacin) or an aminoglycoside co-administered with a macrolide (Table 8 (Tab. 8)). An essential differential therapeutic criterion is antibiotic pre-treatment, which then requires a change of the drug group. Ceftazidime is also active against *Pseudomonas aeruginosa* but has inadequate activity against *Streptococcus pneumoniae* and *Staphylococcus aureus* compared to cefepime. After clinical improvement and/or pathogen identification with susceptibility testing, a de-escalation to beta-lactam or fluoroquinolone monotherapy should generally be performed, if possible taking into account the antibiotic susceptibility testing. Aminoglycosides should generally not be given for more than 3 days due to increased toxicity. In case of previous antibiotic treatment within the last 3 months, a change of the last used drug group is recommended. This applies in particular to previous treatment with fluoroquinolone.

### Duration of treatment with severe CAP

In patients with a good response without complications, a treatment duration of 7 days or at least 2 days after reaching clinical stability is recommended. If infection with *Pseudomonas aeruginosa* is proven, the duration of therapy should be 8–15 days. sCAP with *Staphylococcus* *aureus* may also require a longer duration of treatment.

## Nosocomial pneumonia

Nosocomial pneumonia is a hospital acquired infection that occurs at the earliest 48 hours after hospital admission and was not yet in incubation at the time of in-patient admission. Pneumonia which occur in the first weeks after discharge from hospital are also considered nosocomial infections as colonization with hospital pathogens can often be proven. However, there is currently no generally accepted time frame for this. 

In the US and Europe, pneumonia is the second most common nosocomial infection; in intensive care medicine it is the leading one. The likelihood of developing pneumonia increases with the length of stay in the intensive care ward and during the first 7–10 days of mechanical ventilation, after which it decreases again [16], [17]. In particular, infections with multidrug-resistant bacteria show an unfavorable prognosis. Early and effective treatment for nosocomial pneumonia can be crucial in reducing morbidity and mortality [18], [19], [20].

The pathogen spectrum of nosocomial pneumonia is very broad, with bacterial pathogens dominating and in many cases as a polymicrobial bacterial infection. Fungi and viruses (with the exception of nosocomial influenza infection during the season) are rarely found to cause nosocomial pneumonia in immunocompetent patients. In nosocomial pneumonia there is a shift in the spectrum of pathogens to aerobic and facultative anaerobic Gram-negative rod bacteria such as *Pseudomonas aeruginosa*, Enterobacteriaceae (*Escherichia coli*, *Klebsiella* spp. and *Enterobacter* spp.), *Haemophilus influenzae*, *Acinetobacter baumannii* and *Stenotrophomonas maltophilia*. The main Gram-positive pathogens of nosocomial pneumonia are *Staphylococcus aureus* and *Streptococcus pneumoniae*. 

For the selection of the calculated initial treatment of nosocomial pneumonia, the S3 guideline “Nosocomial Pneumonia”, updated in 2017, takes into account the expected pathogen spectrum and individual risk factors for multidrug-resistant pathogens, which are not weighted individually. The recommended treatment algorithms take into account both disease severity (sepsis-associated organ dysfunction or invasive ventilation) and risk of MDROs. In patients without risk of MDROs, monotherapy is generally recommended regardless of the severity of the disease. In patients with risk of MDROs without sepsis-associated organ dysfunction and without invasive mechanical ventilation, initial monotherapy with a Pseudomonas-active beta-lactam should be preferred. Ceftazidime is not suitable for calculated monotherapy as this drug does not have sufficient activity in the Gram-positive range (Staphylococcus aureus, Streptococcus pneumoniae). A calculated combination therapy should be reserved for patients at increased risk of multidrug-resistant pathogens and sepsis-associated organ dysfunction or invasive ventilation. In addition, there is a simple scoring system which was introduced in 2003 and used previously in PEG recommendations for the identification of patients at increased risk of infection with multidrug-resistant pathogens. In principle, the same risk factors are used here as in the S3 guideline but the risk factors are weighted according to a scoring scheme. Accordingly, three treatment algorithms are presented – monotherapy in patients without risk factors for MDROs (in line with the S3 guideline), monotherapy in patients at low risk for MDROs and combination therapy in patients at high risk for MDROs. The score allows a more graduated risk assessment so that fewer patients will be stratified to the combination therapy group. The scientific basis for these recommendations is based on evidence of varying degrees and often reflects the opinions of experts. So far, there is a retrospective evaluation of the score [21]; a prospective evaluation has not yet been carried out. Due to this weakness of evidence, the score has not been included in the S3 guideline. However, in the context of this S2k Guideline, which was created on the basis of consensus-building, this score should still be used.

To begin with, the S3 guideline: according to the S3 guideline for nosocomial pneumonia, the choice of the initial antimicrobial treatment should be calculated based on the expected pathogen spectrum. Individual risk factors for multidrug-resistant pathogens (MDROs) should be taken into account (see Table 9 (Tab. 9)) as well as the local resistance situation [3]. 

Independent of the severity, monotherapy is indicated when there is no increased risk of infection by multidrug-resistant pathogens. Even in the presence of MDRO risk factors, in less severe cases without sepsis-associated organ dysfunction and without invasive ventilation, an initial monotherapy with a *Pseudomonas*-active drug depending on the individual patient factors and local resistance spectrum is an adequate treatment option. Initial combination therapy should generally only be prescribed if there are risk factors for the presence of multi-resistant pathogens combined with a life-threatening infection (sepsis-associated organ dysfunction or invasive mechanical ventilation) to minimize the risk of inadequate initial therapy in these at-risk patients. The initial combination therapy has to be re-evaluated after 2–3 days of treatment [20], [22], [23]. If a susceptible pathogen or clinical stabilization is detected, treatment should be changed to monotherapy, ideally with the beta-lactam or alternatively fluoroquinolone contained in the initial treatment. An initial calculated treatment against MRSA should be terminated if this pathogen has not been detected. The rapid onset of adequate antimicrobial therapy in sufficiently high doses is critical to successful treatment [24], [25]. The duration of treatment should not exceed 7–10 days [3], [26]. Bacteremic *Staphylococcus* *aureus* pneumonia should usually be treated for at least 14 days.

### Initial calculated treatment in patients with nosocomial pneumonia without increased risk of infection by multidrug-resistant pathogens 

The pathogen spectrum of this patient group largely corresponds to the endogenous flora of the upper airways which the patient has brought from his living environment. This includes *Streptococcus* *pneumoniae*, methicillin-susceptible *Staphylococcus* *aureus*, *Haemophilus* *influenzae* and other Gram-negative pathogens. Multidrug-resistant bacteria play a minor role as long as no respective risk factors are present.

For treatment of these patients, group 3a cephalosporins, aminopenicillin/beta-lactamase inhibitor combinations or pneumococcal fluoroquinolones are recommended (see Table 10 (Tab. 10)). It should be noted, however, that when using group 3 cephalosporins, there may be an increased selection of vancomycin-resistant enterococci (VRE), ESBL-producing Enterobacteriaceae, and beta-lactam antibiotic-resistant *Acinetobacter* spp. [27]. Use of fluoroquinolones should also be prescribed with caution because of the frequent selection of resistant strains [28].

The initial treatment should always be parenteral and oral therapy may be used after clinical improvement. It is also possible to start treatment initially with fluoroquinolones orally if gastrointestinal absorption rates are normal and if patient cooperation is guaranteed.

### Initial calculated treatment in patients with nosocomial pneumonia with increased risk of infection by multidrug-resistant pathogens 

In addition to the previously mentioned common pathogens, in this group of patients *Enterobacter* spp., *Serratia* spp., *Citrobacter* spp. and *Pseudomonas*, *Acinetobacter* spp., *Stenotrophomonas maltophilia* and anaerobes may be encountered. For treatment, therefore, antibiotics should be used that cover these pathogens in their spectrum. The choice is therefore acylaminopenicillins/ beta-lactamase inhibitor (BLI) or *Pseudomonas*-effective carbapenems or cephalosporins, respectively. In patients with life-threatening infection (sepsis-associated organ dysfunction or invasive mechanical ventilation), initial treatment should be combined with a *Pseudomonas*-active fluoroquinolone or an aminoglycoside to increase the coverage (see Table 11(Tab. 11)). Ceftazidime should only be used in combination, because of its insufficient efficacy against Gram-positive cocci. 

Since the end of 2014, ceftobiprole medocaril has been approved in Germany for the treatment of nosocomial pneumonia in adults (with the exception of ventilator associated pneumonia). As a broad-spectrum antibiotic, it belongs to group 5 of the cephalosporins, which is characterized by bactericidal activity against Gram-positive and Gram-negative pathogens. Its in vitro efficacy against MRSA and *Pseudomonas aeruginosa* and Enterobacteriaceae predestines this drug for the indication “nosocomial pneumonia”. Based on clinical data of a phase III trial demonstrating inferiority in ventilator-associated pneumonia, which may indicate underdose of the drug for this indication, ceftobiprole is indicated in nosocomial pneumonia (other than VAP) when risk factors for MRSA and *Pseudomonas aeruginosa* (dialysis, prior treatment with antibiotics, known MRSA colonization or individual *Pseudomonas aeruginosa* identification) are present. The efficacy of ceftobiprole against *Pseudomonas aeruginosa* is comparable to that of ceftazidime. However the inadequate antibacterial activity against non-fermenters (*Acinetobacter* spp., *Burkholderia cepacia* complex, *Stenotrophomonas maltophilia*) as well as against ESBL-producing Enterobacteriaceae, argues against broad empirical therapeutic use on intensive care wards.

With ceftolozane, another *Pseudomonas*-active beta-lactam is available, which given in fixed combination with tazobactam has an extended spectrum of action compared to ceftazidime or cefepime, including ESBL-producing Enterobacteriaceae. Thus, the drug is of particular interest for the treatment of nosocomial pneumonia in the presence of risk factors for *Pseudomonas aeruginosa* and multidrug-resistant Enterobacteriaceae. However, ceftolozane/tazobactam also is inadequately active against *Streptococcus pneumoniae* and shows no activity against *Staphylococcus aureus*. Although ceftolozane/ tazobactam is currently being studied for the indication pneumonia, it has not yet been approved for this indication. 

In the following, the score configured in 2003 by the PEG and the German Society of Pneumology (DGP), which assigns a weighting of the individual risk factors and thus may be used as an alternative to the recommendations of the S3 guideline, to estimate of the risk of an infection caused by multi-resistant pathogens.

The score recommends calculated initial antimicrobial treatment based on the assignment to defined risk groups with a characteristic pathogen spectrum. Each of the three groups has its specific risk profile, which is represented by a corresponding point score, summarizing the presence of individual risk factors. In this score, each risk factor is subject to a different weighting of 1 to 4 points (Table 12 (Tab. 12)). Each risk factors has a varying impact on the severity of the disease and the expected spectrum of pathogens.

In the context of this point score, fosfomycin is recommended as combination partner in addition to a fluoroquinolone or a aminoglycoside, which are also recommended in the S3 guideline. This (recommendation) is based on the fact that in some cases fluoroquinolones may no longer be considered as safe combination partners, depending on the local resistance situation. Fosfomycin shows a high ability to penetrate into lung tissue and the susceptibility of MRSA continues to be regarded as very favorable. However, there is no data from prospective randomized clinical trials, so it was not possible to include fosfomycin into the S3 guideline due to a lack of evidence. Nevertheless, it may be incorporated here as a combination partner due to its aforementioned advantages. 

## MRSA pneumonia

From a clinical point of view, most data on the treatment of MRSA pneumonia are available for linezolid and the glycopeptides. In a post-hoc analysis of two prospective studies, linezolid was statistically significantly more advantageous than vancomycin [29] but it was not superior to vancomycin regarding the primary endpoint in another clinical trial [30]. In the ZEPHyR study, a prospective randomized trial comparing linezolid with vancomycin, a better clinical response was observed to linezolid, but mortality did not differ significantly [31].

The key disadvantage of vancomycin is its poor penetration into the lung (11% of the plasma level), which could be partially compensated by combination with a tissue penetrating MRSA-active drug (fosfomycin, rifampicin). A randomized clinical trial has shown that the combination of vancomycin and rifampicin significantly improves the clinical cure rate compared to vancomycin monotherapy in MRSA pneumonia [32]. Other MRSA-active drugs are teicoplanin, tedizolid, ceftaroline and ceftobiprole, as well as the fosfomycin and rifampicin, which are both given as part of a combination therapy. Daptomycin is generally not suitable for the treatment of pulmonary infections, as it is inactivated by surfactant. The targeted treatment should be based on susceptibility testing. If there is no evidence of MRSA, MRSA-effective treatment should be discontinued.

Since linezolid, like vancomycin, exclusively covers Gram-positive pathogens, monotherapy with the drug should only be prescribed if simultaneous infection with Gram-negative pathogens has been ruled out.

### Adjustment of the initial treatment – targeted treatment after identification of specific pathogens/MDROs 

If a MDRO is detected, the calculated initial treatment should be adjusted according to the result of susceptibility testing. As a rule, monotherapy is possible with targeted treatment. Treatment options for defined MDROs are shown below:

- **MRSA strains**: Anti-infectives suitable for monotherapy include vancomycin, teicoplanin, ceftobiprole and linezolid. In severe diseases, the combination of vancomycin with rifampicin is another option.
- **MDRO *Pseudomonas*** ***aeruginosa****:* Effective treatment options include ceftazidime, cefepime, ceftolozane (only in fixed combination with tazobactam; currently not approved for the treatment of pneumonia), piperacillin, imipenem and meropenem, as well as ciprofloxacin and levofloxacin. The combination of a *Pseudomonas*-active beta-lactam antibiotic with an aminoglycoside (gentamicin, tobramycin, amikacin) or a fluoroquinolone should be considered on a case-by-case basis (severe infections). However, superiority to monotherapy is not proven. Despite confirmed in vitro efficacy, fluoroquinolones as well as beta-lactams showed lower eradication rates for *Pseudomonas aeruginosa* than for enterobacteria and *Staphylococcus aureus* [33], [34], [35]. Various pharmacokinetic studies have shown that in a large proportion of patients with severe sepsis and septic shock the standard doses of most antibiotics result in serum concentrations below the required PK/PD indices for the particular antibiotic [36], [37]. This applies in particular to fluoroquinolones, as the PK/PD parameter (AUC/MIC≥125) required for *Pseudomonas* *aeruginosa* is not reliably achieved even in patients on normal wards [38], [39], [40], [41]. Therefore, in the case of targeted monotherapy of *Pseudomonas aeruginosa* pneumonia with levofloxacin or ciprofloxacin, a high dose should be selected (for instance 2x 500 mg levofloxacin or 2x 750 mg ciprofloxacin p.o. or 3x 400 mg ciprofloxacin i.v.) and the patient should be monitored closely for treatment failure. In case of resistance to all standard drugs, treatment with colistin is indicated; combination therapy should be the aim, if possible after consultation with an infectious disease specialist/microbiologist.
- **ESBL strains**: Carbapenems are effective. In case of additional resistance to carbapenems, colistin is used, if possible in combination therapy after consultation with an infectious disease specialist/microbiologist. Another option would be ceftazidime/avibactam.
- **Stenotrophomonas maltophilia**: Cotrimoxazole is indicated, when in vitro susceptibility is proven. In the case of cotrimoxazole resistance, a susceptibility test for ceftazidime, moxifloxacin, levofloxacin and tigecycline (not approved for the treatment of pneumonia) should be carried out and one of these drugs should be used. Prior to this, the clinical relevance of the isolate must be checked.
- **Acinetobacter** spp.: Imipenem or meropenem are effective most frequently. In infections caused by carbapenemase-producing strains colistin is indicated, if possible in combination with another in vitro active drug. Tigecycline is an additional option for salvage therapy but is not approved for the treatment of pneumonia.

## Aspiration pneumonia and lung abscess

Aspiration pneumonias divided into chronic recurrent subtle aspirations and acute aspirations of gastric contents.

- Pathogen detection is difficult.
- A polymicrobial etiology (aerobic and anaerobic pathogens) is common.
- In the case of aspirations that occurred outside the hospital, Gram-positive pathogens are more likely.
- In multimorbid patients with multiple hospital stays and with a history of antimicrobial treatments, Gram-negative pathogens or polymicrobial infections are usually the cause, in part with involvement of anaerobes.

The pathogenesis of primary lung abscesses is based on the aspiration and corresponding virulence of the pathogens or reduced immunocompetence of the patient. Risk factor for aspiration include

- pre-existing diseases of the CNS,
- intoxications,
- dysphagia and/or
- esophageal pathologies.

Secondary lung abscesses are caused by

- bronchial obstruction due to neoplasia,
- bronchial obstruction due to foreign bodies with poststenotic pneumonia,
- liquefaction/cavitation,
- superinfection of infarct pneumonia and
- rarely also in bacteremia.

Mixed bacterial infections predominate and obligate anaerobes are detected in 20–90% of cases. In a German study, *Staphylococcus aureus* was identified as the most common pathogen in aspiration pneumonia and lung abscesses [42].

Previous aspiration is a risk factor for Enterobacteriaceae infections. Since an additional etiological role of anaerobic bacteria in aspiration pneumonia cannot be excluded and the majority of anaerobes produce beta-lactamases, a penicillin derivative should be combined with a beta-lactamase inhibitor (ampicillin/sulbactam, amoxicillin/ clavulanic acid). Alternatively, a combination of a cephalosporin group 3a (cefotaxime, ceftriaxone) with clindamycin, or monotherapy with moxifloxacin or ertapenem may be used. While in an uncomplicated aspiration pneumonia a treatment duration of 7–10 days is usually sufficient, in cases with abscess formation longer antimicrobial treatment is often required.

## Infections of the pleura

There are only few reliable data available for the empiric treatment of pleural infections. The evidence is based primarily on retrospective studies and expert opinions.

The main goals of treatment of the parapneumonic effusion are control of the infection, drainage of the infected effusion, (re-)expansion of the lungs and prevention of the development of pleural disorders.

The therapeutic basis is sufficient, pathogen-compliant antimicrobial treatment with the aim of controlling the underlying infection. There are no controlled clinical studies on the topic of antibiotic treatment and treatment duration. Empiric antimicrobial treatment should include Gram-positive cocci, Gram-negative pathogens (possibly including *Pseudomonas aeruginosa*) and anaerobes. In order to achieve sufficiently high serum and pleural concentrations, initial parenteral administration is preferred. In general, it should be continued at least until complete drainage of the infected effusion. Longer treatment periods of several weeks are often required. A cornerstone of the treatment of complicated parapneumonic effusion or pleural empyema is the effective and complete drainage of the infected fluid. Please refer to the corresponding guideline [2].

## Notes

This is the fifth chapter of the guideline “Calculated initial parenteral treatment of bacterial infections in adults – update 2018” in the 2^nd^ updated version. The German guideline by the Paul-Ehrlich-Gesellschaft für Chemotherapie e.V. (PEG) has been translated to address an international audience. 

Following the publication of the 1^st^ version of the guideline in German, this dosage suggestion was updated by the working group (Table 5: Oral sequencing therapy in patients with achieved clinical stability and without pathogenesis in CAP): 1–2x 500 mg Levofloxacin p.o. per day INSTEAD OF 2x 500 mg Levofloxacin p.o. per day.

## Competing interests

The authors declare that they have no competing interests. 

## Figures and Tables

**Table 1:**
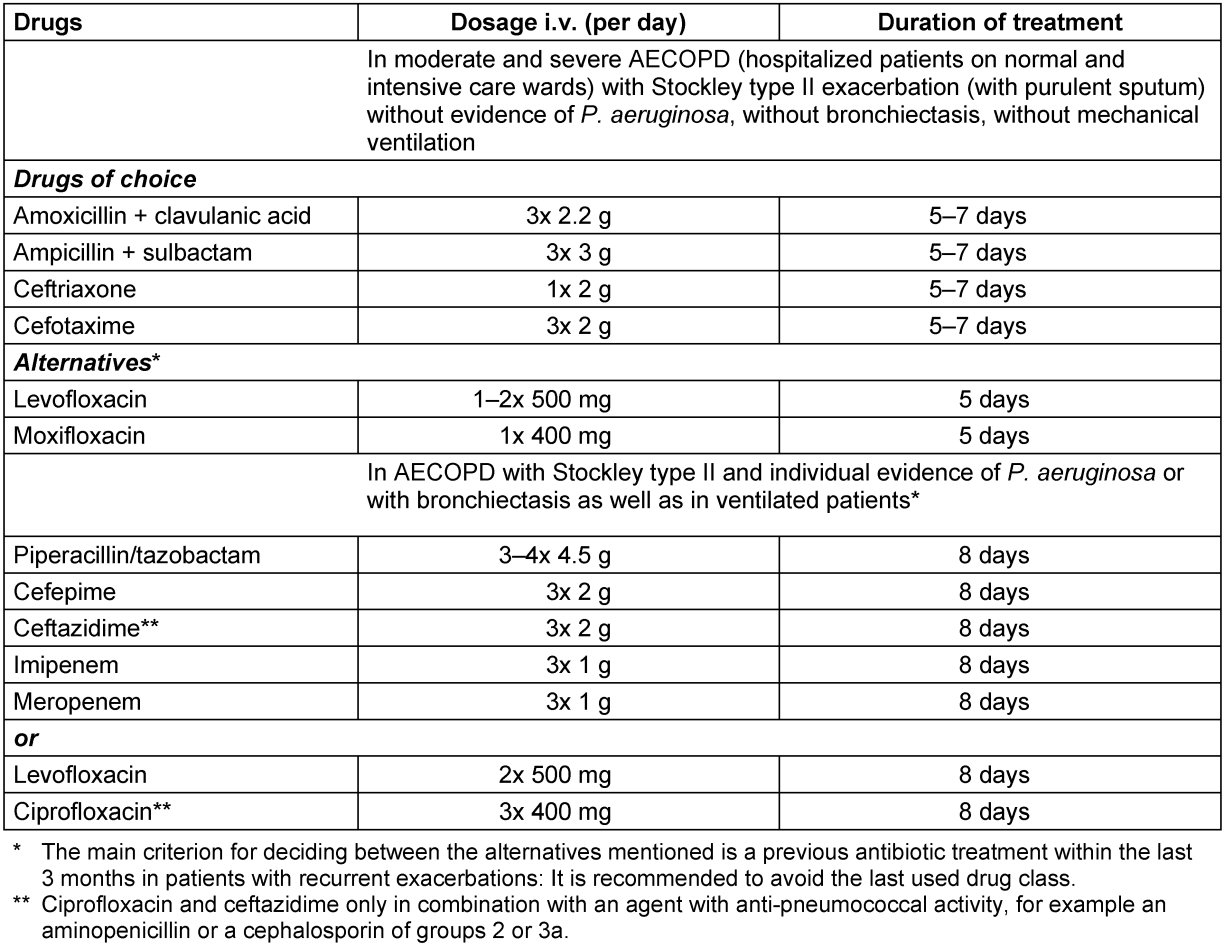
Therapy recommendations for patients with AECOPD in the case of an indication for antibiotic treatment

**Table 2:**
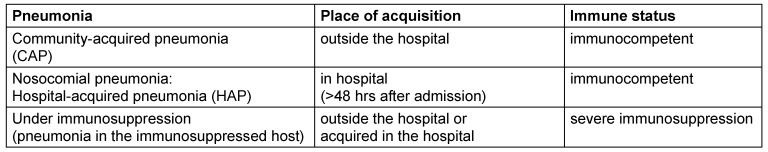
Classification of pneumonia by place of acquisition and immune status of the patient

**Table 3:**
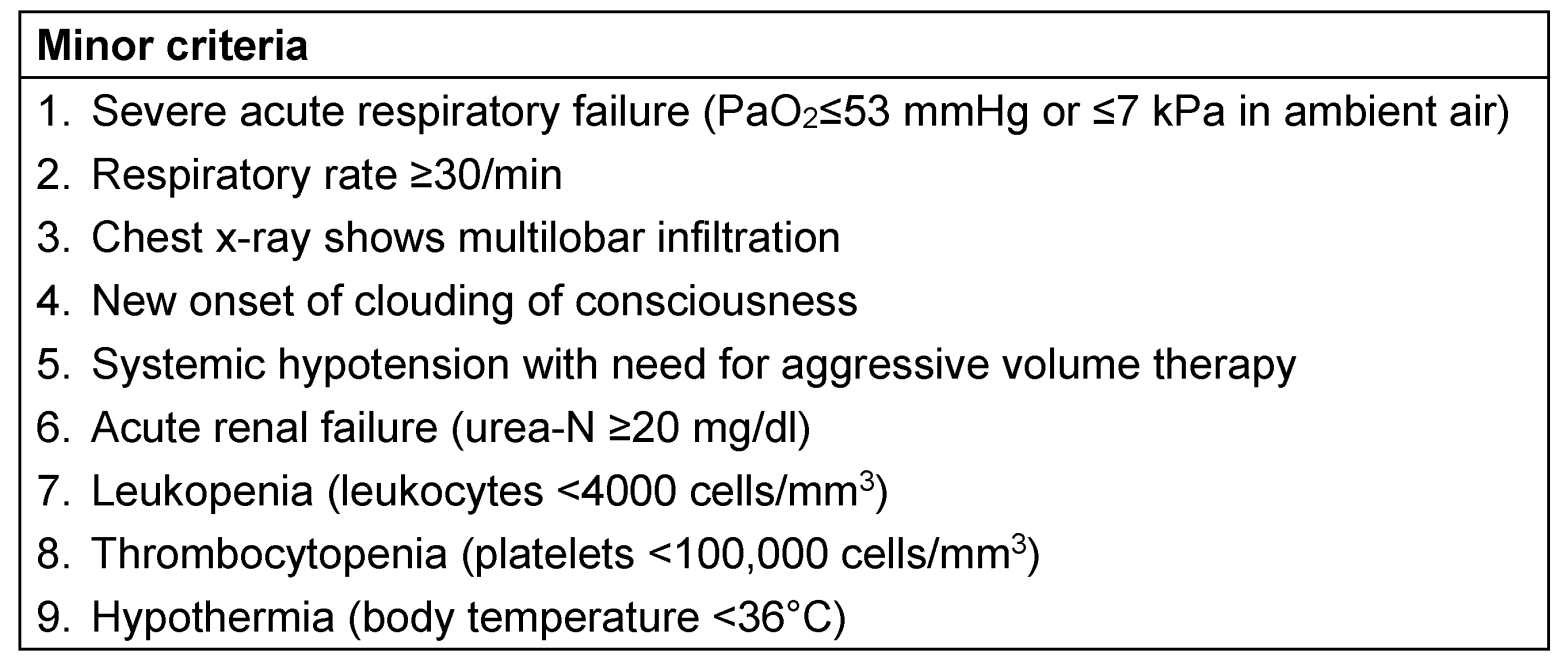
Minor criteria. A high risk for requiring intensive treatment exists when >1 of the 9 minor criteria are present.

**Table 4:**
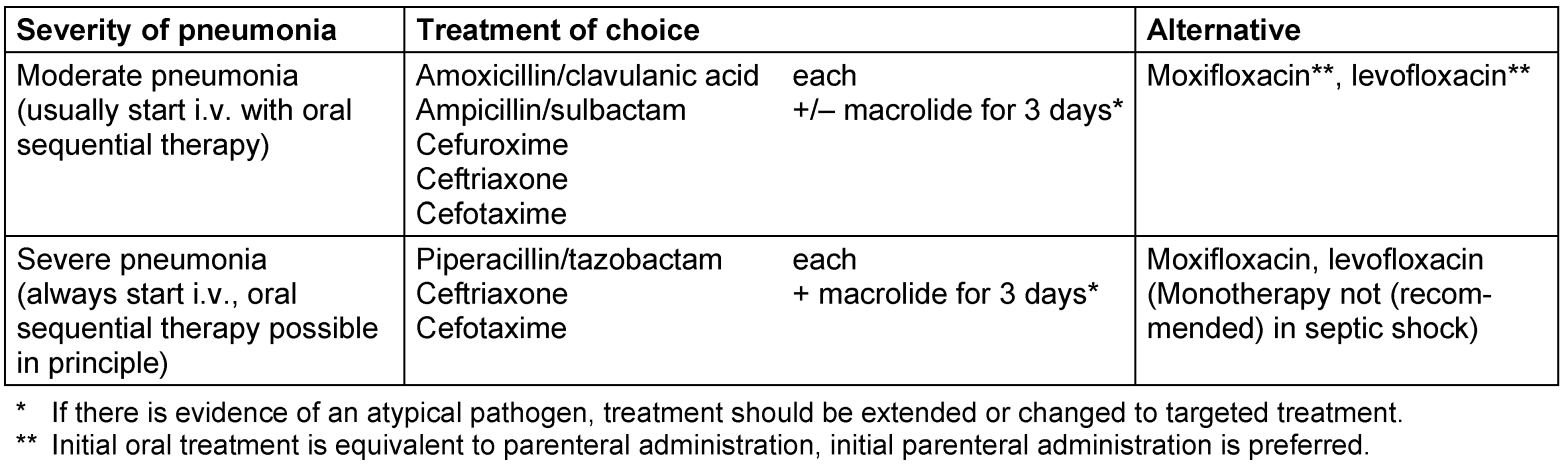
Recommendation for the calculated initial treatment in moderate and severe hospitalized CAP without risk for multi-resistant pathogens (adapted from [2]). For dosage recommendations, see Table 7

**Table 5:**
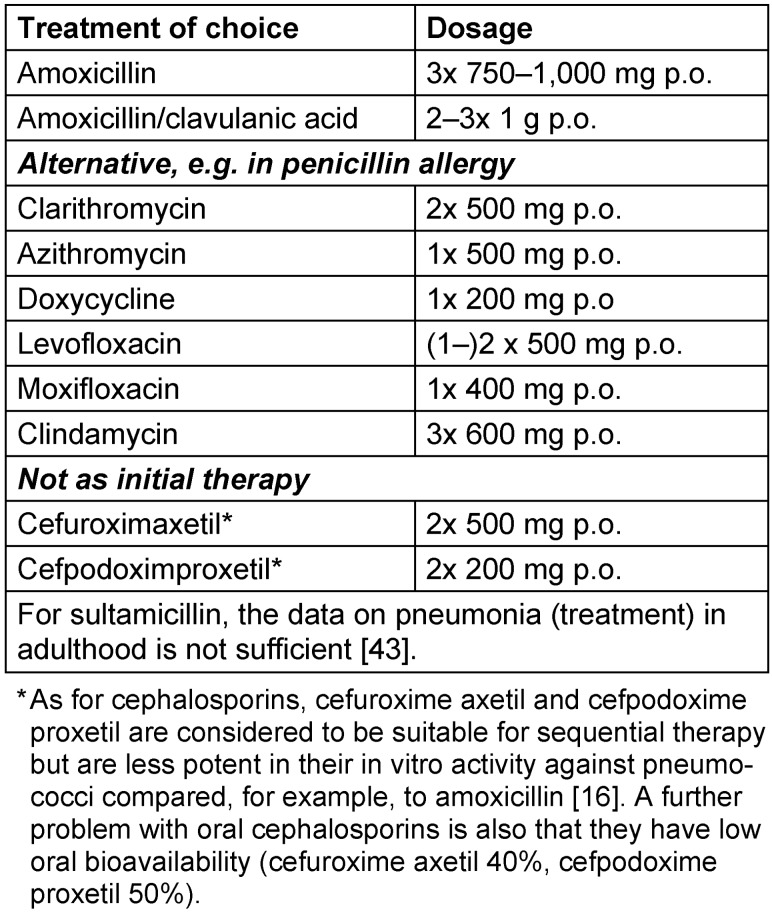
Oral sequential therapy in patients with CAP after achieving clinical stability and without pathogen identification (if the pathogen has been identified: targeted therapy, see Table 6)

**Table 6:**
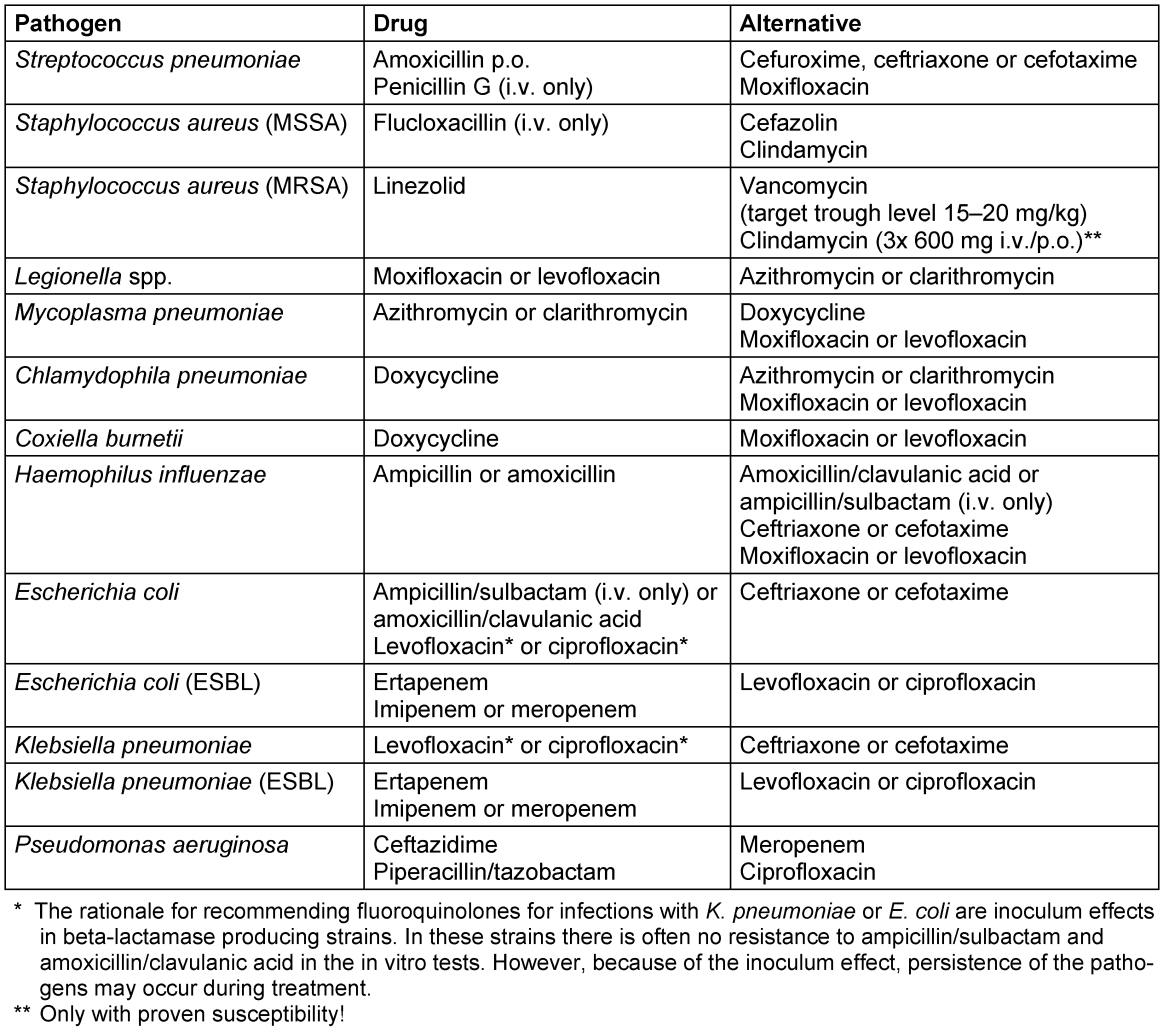
Selected options for targeted antimicrobial treatment of patients with community-acquired pneumonia after pathogen identification and in vitro susceptibility testing (adapted from [2]). Dosages – unless otherwise stated – see Table 7

**Table 7:**
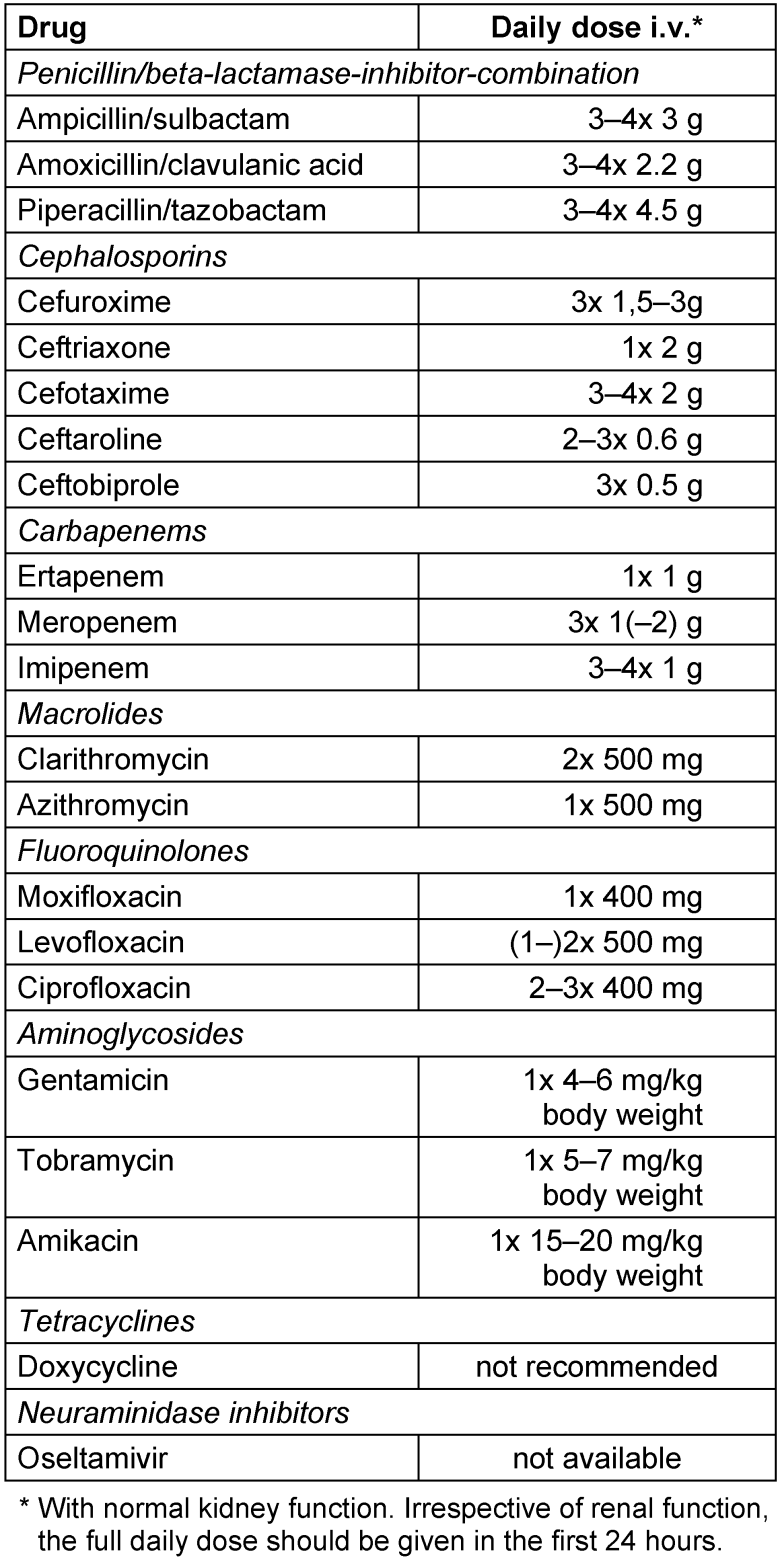
Dosage recommendations for the calculated parenteral antimicrobial initial treatment

**Table 8:**
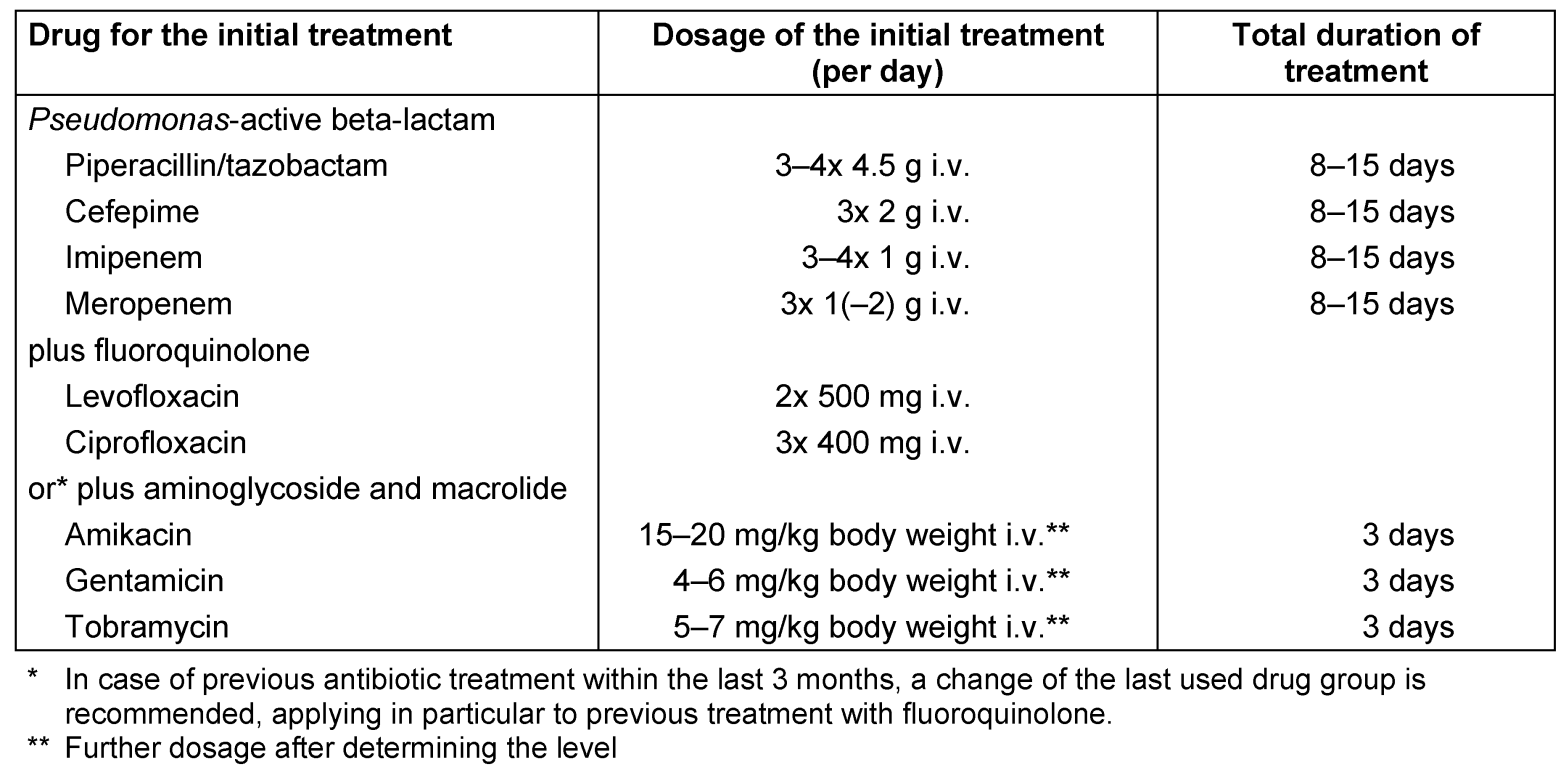
Recommendation for calculated initial parenteral treatment in hospitalized patients with individual risk factors for multidrug-resistant pathogens (MDROs)

**Table 9:**
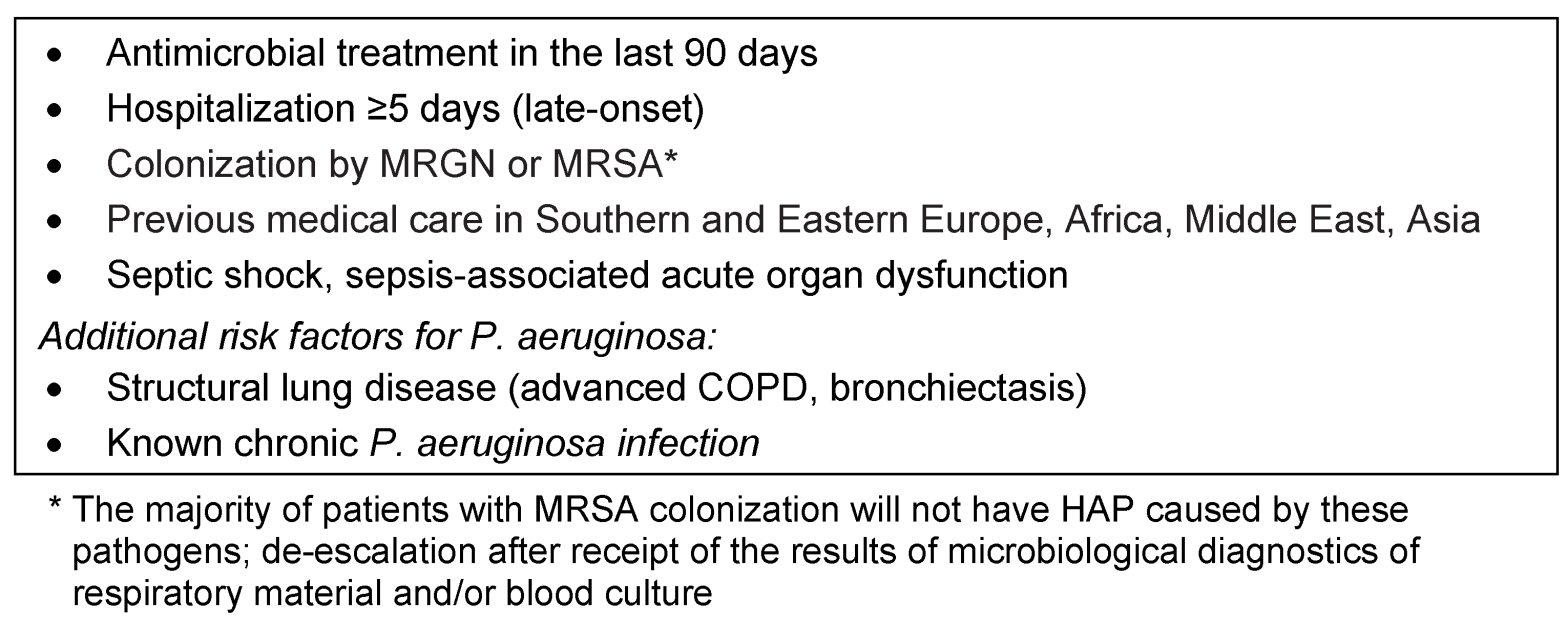
Risk factors for multidrug-resistant pathogens (MDROs) in nosocomial pneumonia (after [3])

**Table 10:**
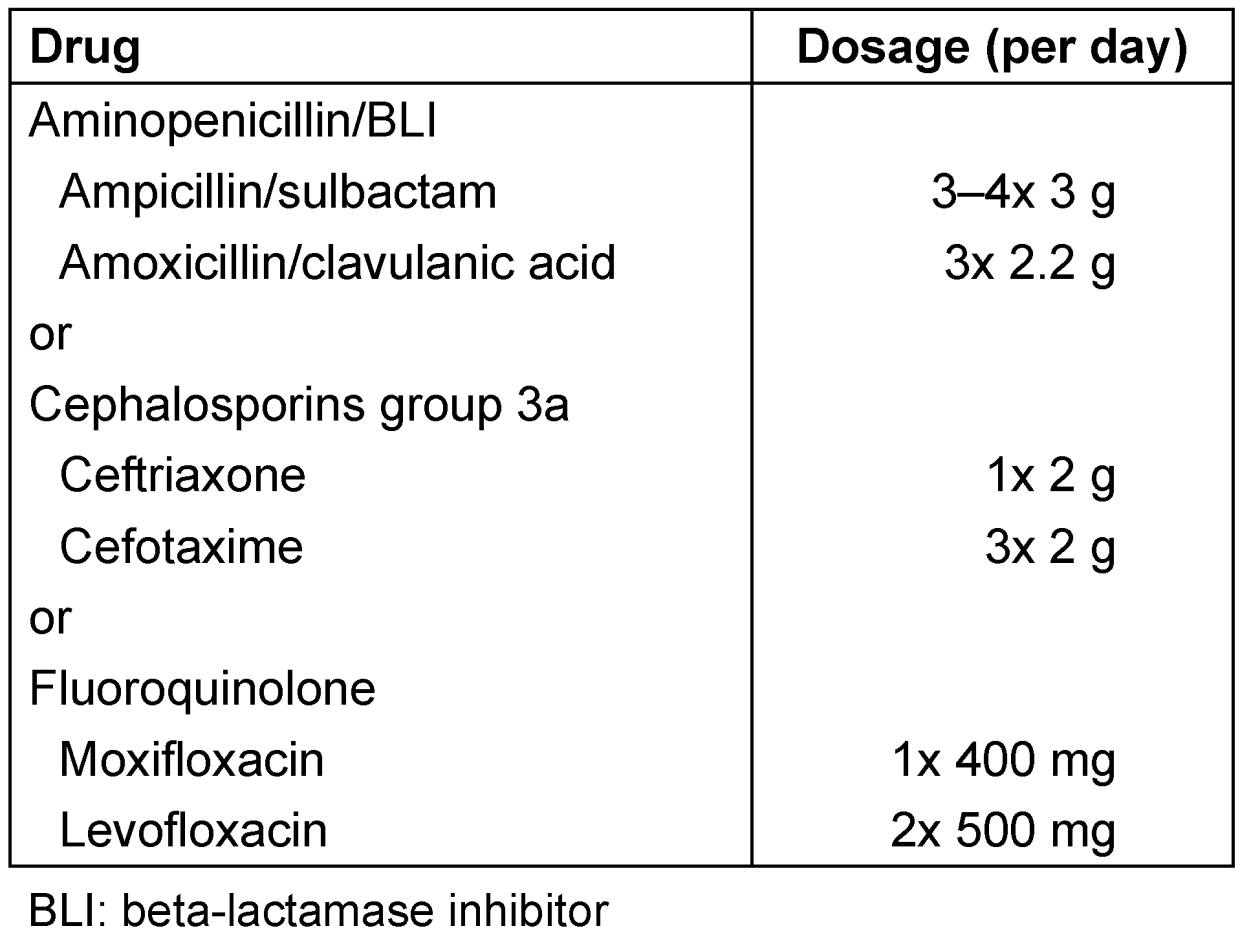
Calculated initial treatment for nosocomial pneumonia without risk factors for multidrug resistant pathogens (adapted from [3])

**Table 11:**
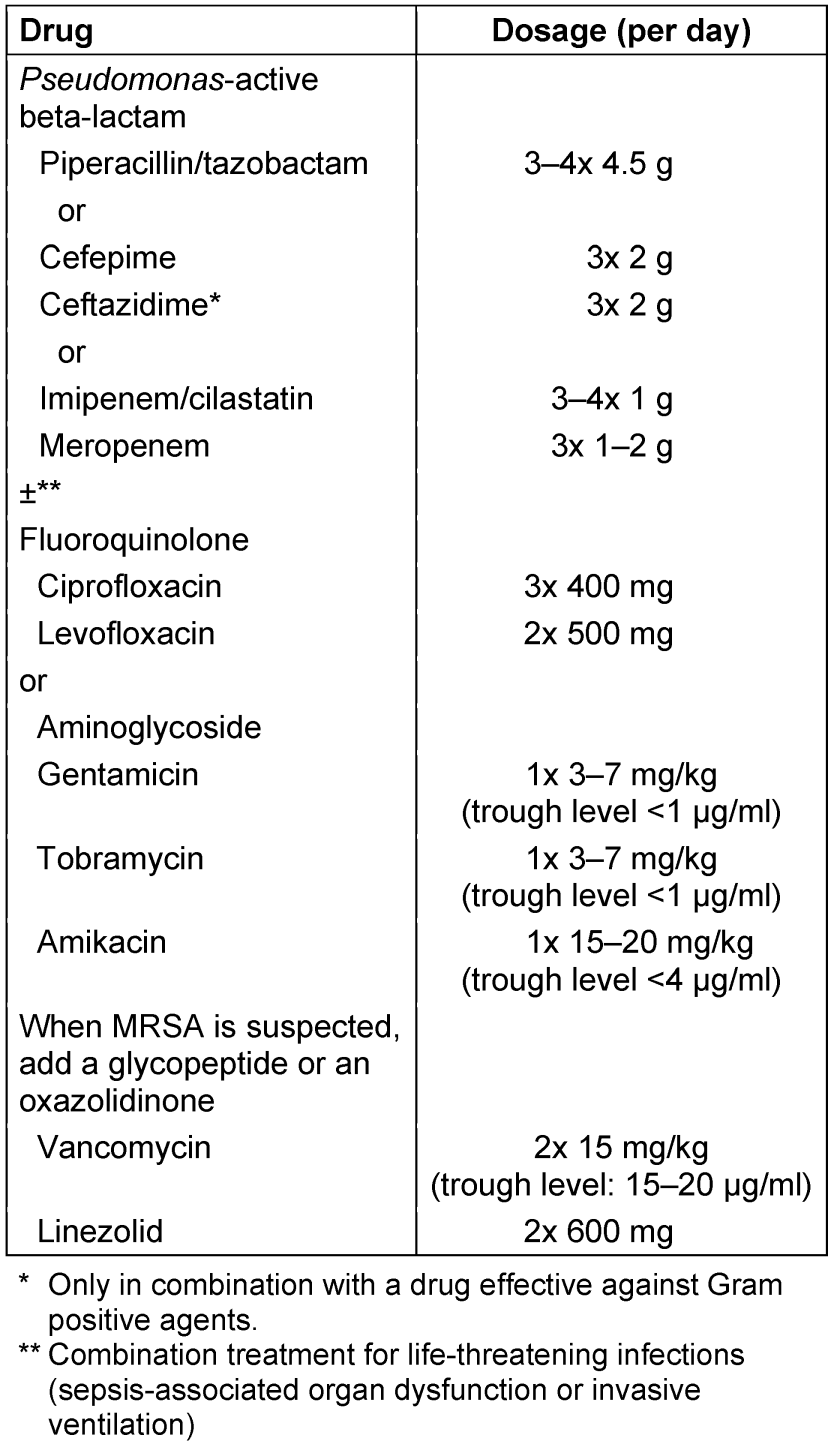
Recommendation for the calculated parenteral initial treatment in patients with nosocomial pneumonia and risk factors for multidrug-resistant pathogens (adapted after [3])

**Table 12:**
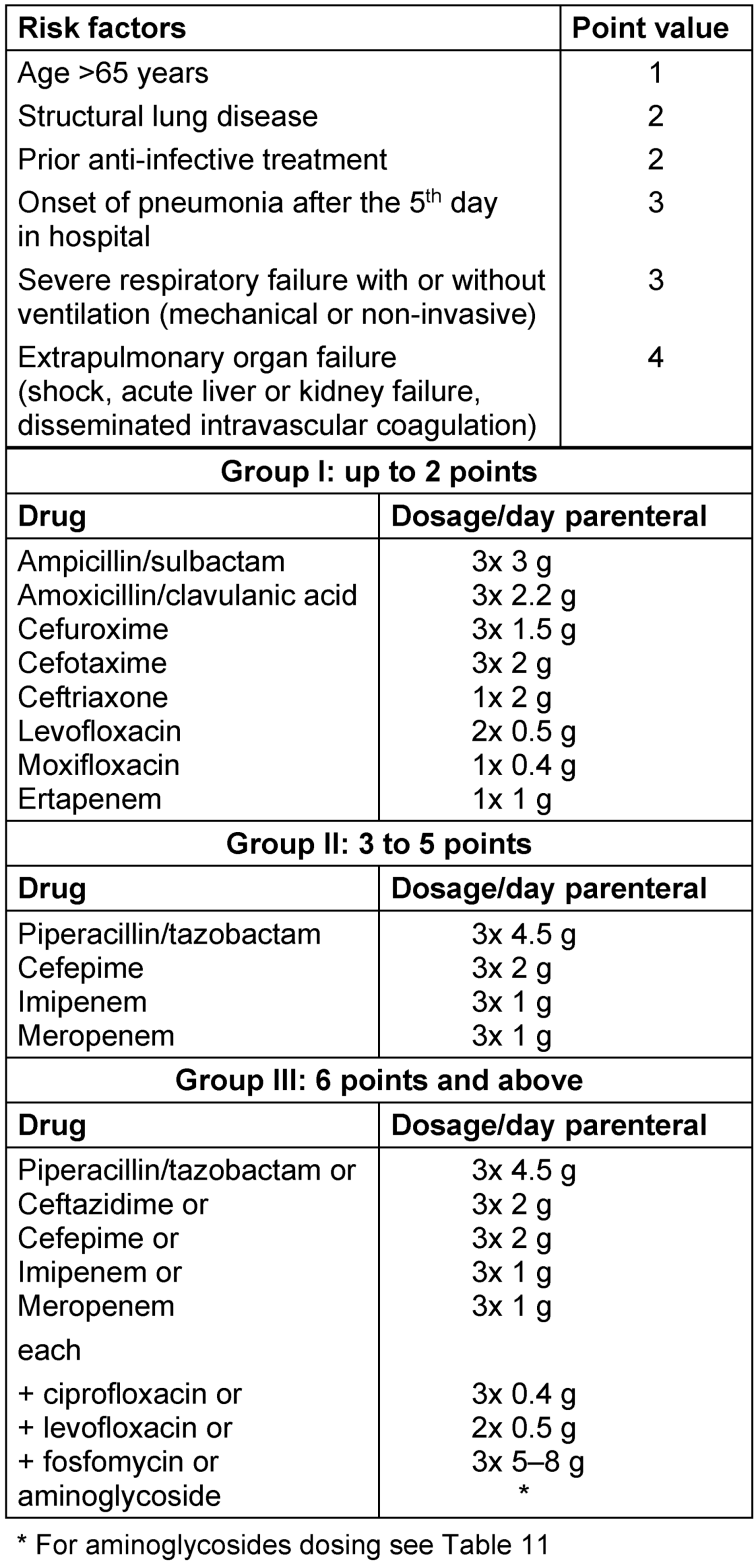
Score assessment of risk factors in patients with nosocomial pneumonia and recommendations for calculated initial treatment
